# Water Fraction Dependence of the Aggregation Behavior of Hydrophobic Fluorescent Solutes in Water–Tetrahydrofuran

**DOI:** 10.1021/acs.jpclett.3c02882

**Published:** 2023-12-07

**Authors:** Hayato Tsuji, Masaki Nakahata, Mafumi Hishida, Hideki Seto, Ryuhei Motokawa, Takeru Inoue, Yasunobu Egawa

**Affiliations:** † Department of Chemistry, Faculty of Science, Kanagawa University, 3-27-1 Rokkaku-bashi, Kanagawa-ku, Yokohama 221-8686, Japan; ‡ Department of Macromolecular Science, Graduate School of Science, Osaka University, 1-1 Machikaneyama-cho, Toyonaka, Osaka 560-0043, Japan; § Department of Chemistry, Faculty of Science, Tokyo University of Science, 1-3 Kagurazaka, Shinjuku, Tokyo 162−8601, Japan; ∥ Institute of Materials Structure Science / J-PARC Center, High Energy Accelerator Research Organization, Tokai, Ibaraki 319-1106, Japan; ⊥ Materials Sciences Research Center, Japan Atomic Energy Agency, Tokai, Ibaraki 319-1195, Japan

## Abstract

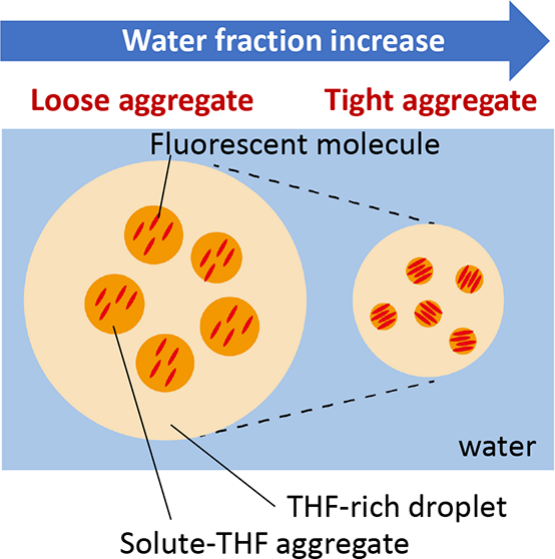

This work investigates the water fraction dependence of the aggregation behavior of hydrophobic solutes in water–tetrahydrofuran (THF) and the elucidation of the role of THF using fluorescence microscopy, dynamic light scattering, neutron and X-ray scattering, and photoluminescence measurements. On the basis of the obtained results, the following model is proposed: hydrophobic molecules are molecularly dispersed in the low-water-content region (10–20 vol %), while they form mesoscopic particles upon increasing the water fraction to ∼30 vol %. This abrupt change is due to the composition fluctuation of the water–THF binary system to form hydrophobic areas in THF, followed by THF-rich droplets where hydrophobic solutes are incorporated and form loose aggregates. Further increasing the water content prompts the desolvation of THF, which decreases the particle size and generates tight aggregates of solute molecules. This model is consistent with the luminescence behavior of the solutes and will be helpful to control the aggregation state of hydrophobic solutes in various applications.

Nano–mesoscopic-scale aggregates of hydrophobic substances can be stably dispersed in an aqueous environment without a surfactant,^1,2^ and such dispersions have attracted substantial attention in a wide range of fields, including colloidal science, geoscience, materials science, and pharmacology. These dispersions are commonly prepared by dissolving a hydrophobic substance in a water-miscible organic solvent, followed by mixing the resulting solution with water. Owing to its ability to dissolve many organic compounds, tetrahydrofuran (THF) is a commonly used amphiphilic solvent, and water–THF binary systems have been structurally characterized exhaustively using diverse physicochemical techniques^3−15^ and computational methods.^16−19^ The aggregation of organic molecules and polymers in THF–water systems has also attracted growing attention in recent years^20−23^ and, through the exploitation of phenomena such as aggregation-induced emission (AIE)^24−27^ and suppression of aggregation-caused fluorescence quenching (ACQ),^28,29^ has found applications in organic electronics^30−34^ as well as luminescence imaging and sensing.^35^ In this context, the structural characterization of aggregates of luminophores in THF–water systems and the control of their formation are crucial to improve their functionality.^36^

Here, we disclose the aggregation behavior of hydrophobic fluorescent molecules in water–THF mixtures in terms of the water fraction dependence and the role of THF as an amphiphilic solvent in such systems using physicochemical techniques, such as fluorescence spectroscopy and microscopy, dynamic light scattering (DLS), small-angle neutron scattering (SANS), and wide-angle X-ray scattering (WAXS), by taking advantage of the distinct observational range of each method. As a hydrophobic fluorescent molecule, Cz-COPV2-BTz-COPV2-Cz^37,38^ (Figure 1a), which is well-soluble in THF and insoluble in water, was selected as a model case because it forms stable aggregates and shows ACQ behavior while keeping a sufficient photoluminescence quantum yield (PLQY) over a broad range of solvent–mixture compositions, which facilitates the measurements. The behavior of other representative hydrophobic fluorescent materials was also examined by DLS, which revealed that their size change showed a similar solvent mixing ratio dependence, thus suggesting that the formation and size dependence of the hydrophobic solute aggregates follow a general mechanism.

**Figure 1:**
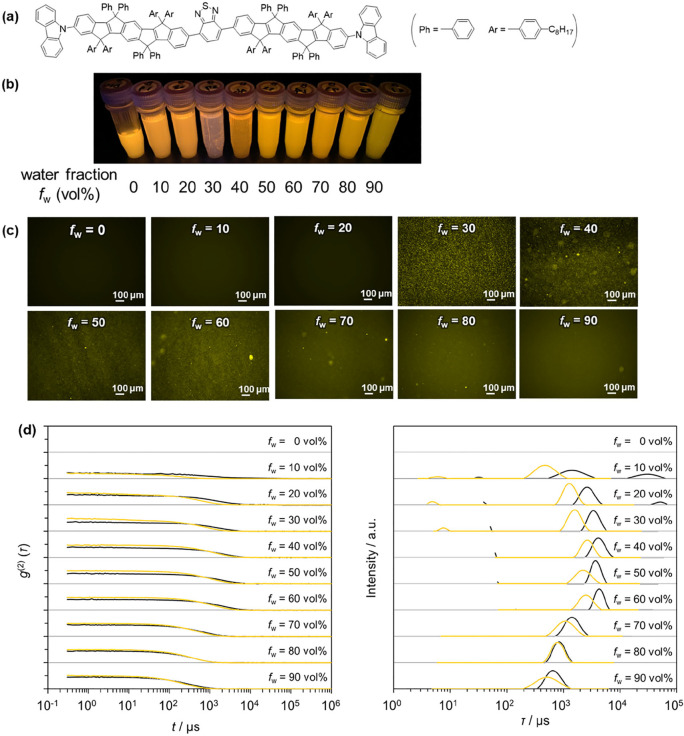
Solutions of Cz-COPV2-BTz-COPV2-Cz at different water-to-THF mixing ratios at room temperature: (a) molecular structure of Cz-COPV2-BTz-COPV2-Cz, (b) photographic image of Cz-COPV2-BTz-COPV2-Cz solutions in water–THF (∼10 μM) under 365 nm ultraviolet (UV) light, (c) fluorescence microscopy images, and (d) DLS data (black, solvents only; yellow, Cz-COPV2-BTz-COPV2-Cz solutions): left, autocorrelation function (ACF); right, relaxation time distribution.

Solutions of Cz-COPV2-BTz-COPV2-Cz in water–THF mixtures with different ratios and concentrations of ∼1 μM, which are commonly used for absorption and emission measurements, were homogeneous irrespective of the solvent mixing ratio, whereas ∼10 μM solutions formed some precipitation at a water fraction (*f*_w_) of 30 vol % (Figure 1b). The fluorescence microscopy images shown in Figure 1c can be roughly classified into three types as the water fraction increases every 10% from *f*_w_ = 0 (pure THF) to 90 vol %: (1) *f*_w_ = 10 and 20 vol %, the THF-rich region, where only dark images were observed under the present measurement conditions (exposure time of 0.25 s); (2) *f*_w_ = 30–60 vol %, the intermediate region, where large fluorescent particles were observed, which indicates the formation of microscopically observable particles in which Cz-COPV2-BTz-COPV2-Cz is dispersed; and (3) *f*_w_ ≥ 70 vol %, the water-rich region, where the number of observable spots decreased and the entire images glowed with the fluorescent color of Cz-COPV2-BTz-COPV2-Cz.

DLS measurements provided quantitative insights into the relaxation time (τ) change of the particles (yellow curves in Figure 1d), whose behavior was similar to that of previously reported fluorescent molecules.^24^ Thus, in the THF-rich region, where dark images were obtained by fluorescent microscopy, the fluorescent dye is most likely molecularly dispersed in the THF-rich medium. In the intermediate region, particles with relaxation times on the order of milliseconds that contain the fluorescence dye are formed, resulting in distinct bright spots in the fluorescence microscopy images. In the water-rich region (*f*_w_ > 60 vol %), the relaxation time of the particles decreases, suggesting the decrease of their size, until they become too small to be observed by fluorescence microscopy but remain as the fluorescent background.

Interestingly, albeit with a quantitative difference, the trend of the water fraction dependence of the relaxation time of the particle in the solutions is qualitatively very similar to that observed in pristine water–THF binary systems (black curves in Figure 1d). It should also be noted here that these trends were also observed for the water–THF–solute ternary system using typical fluorescent molecules showing ACQ or AIE behavior (Figure S1 of the Supporting Information). These results suggest that the particles formed in the water–THF binary system exert a substantial effect on the formation of solute aggregates in water–THF media, in such a way that THF-rich particles provide a mesoscopic-scale field for hydrophobic solute molecules to distribute.

SANS measurements were performed to further investigate the formation of aggregates of Cz-COPV2-BTz-COPV2-Cz and THF and their solvent ratio dependence. In the mixtures of Cz-COPV2-BTz-COPV2-Cz, deuterated water (D_2_O), and THF, the neutron scattering length densities of Cz-COPV2-BTz-COPV2-Cz and THF are comparable and different from that of D_2_O. Thus, the SANS profiles of these mixtures are mainly due to the distribution of THF within D_2_O (Figure 2a). The series of profiles indicates that THF molecules do not form recognizable structures at *f*_w_ ≤ 40 vol % because they are almost flat in the observed range, except for the low-*q* region, where the structure of Cz-COPV2-BTz-COPV2-Cz is likely seen (*vide infra*). Meanwhile, clusters of THF exist at *f*_w_ ≥ 50 vol % in this range.

**Figure 2:**
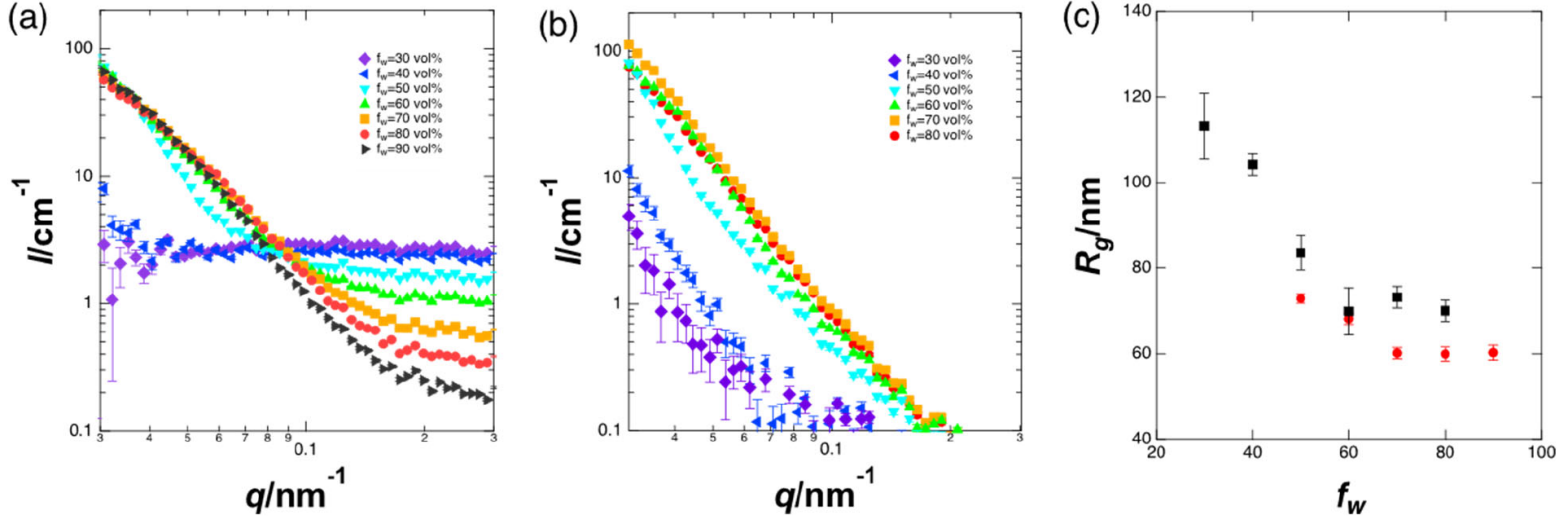
SANS profiles of mixtures of (a) Cz-COPV2-BTz-COPV2-Cz, D_2_O, and THF and (b) Cz-COPV2-BTz-COPV2-Cz, D_2_O, and THF-*d*_8_ at 25 °C. The vertical axes indicate absolute intensity, while the horizontal axes represent the momentum transfer (*q*). (c) Radius of gyration (*R*_g_) of the aggregates of Cz-COPV2-BTz-COPV2-Cz and THF (red circles) estimated from panel a and those of Cz-COPV2-BTz-COPV2-Cz (black squares) estimated from panel b.

The distribution of Cz-COPV2-BTz-COPV2-Cz molecules was obtained from the SANS profiles using a mixture of Cz-COPV2-BTz-COPV2-Cz, D_2_O, and THF-*d*_8_ (Figure 2b) because the scattering length density of deuterated THF (THF-*d*_8_) is comparable to that of D_2_O but different from that of Cz-COPV2-BTz-COPV2-Cz. Interestingly, molecules of Cz-COPV2-BTz-COPV2-Cz already form aggregates at *f*_w_ = 30–40 vol %, where a significant aggregation of THF cannot be expected.

The size of the aggregates was estimated using the Guinier approximation, which affords the radius of gyration (*R*_g_) of the aggregates for *q* → 0. In both systems, the size of the aggregates decreases with increasing *f*_w_ values (Figure 2c). The sizes of the aggregates in COPV2-BTz-COPV2-Cz/THF/D_2_O and COPV2-BTz-COPV2-Cz/THF-*d*_8_/D_2_O are comparable for each solvent ratio at *f*_w_ ≥ 50 vol %, which suggests that the molecules of THF and Cz-COPV2-BTz-COPV2-Cz are almost evenly distributed within the aggregates in this region.

WAXS measurements of Cz-COPV2-BTz-COPV2-Cz in water–THF afforded further information about the water fraction dependence of the aggregation of solute molecules. As shown in Figure 3a, a scattering peak was found at ∼1.4 Å^–1^ (corresponding to 4.5 Å) in the THF-rich region. With increasing water ratio, the peak shifts to the high-*q* region (*q* = 1.7 Å^–1^, corresponding to 3.7 Å), indicating that solute molecules aggregate more tightly in the water-rich region. The fact that the distance is similar to that of π–π stacking interactions (3.4 Å) indicates that the Cz-COPV2-BTz-COPV2-Cz molecules are almost completely stacked onto each other in water-rich environments.

**Figure 3:**
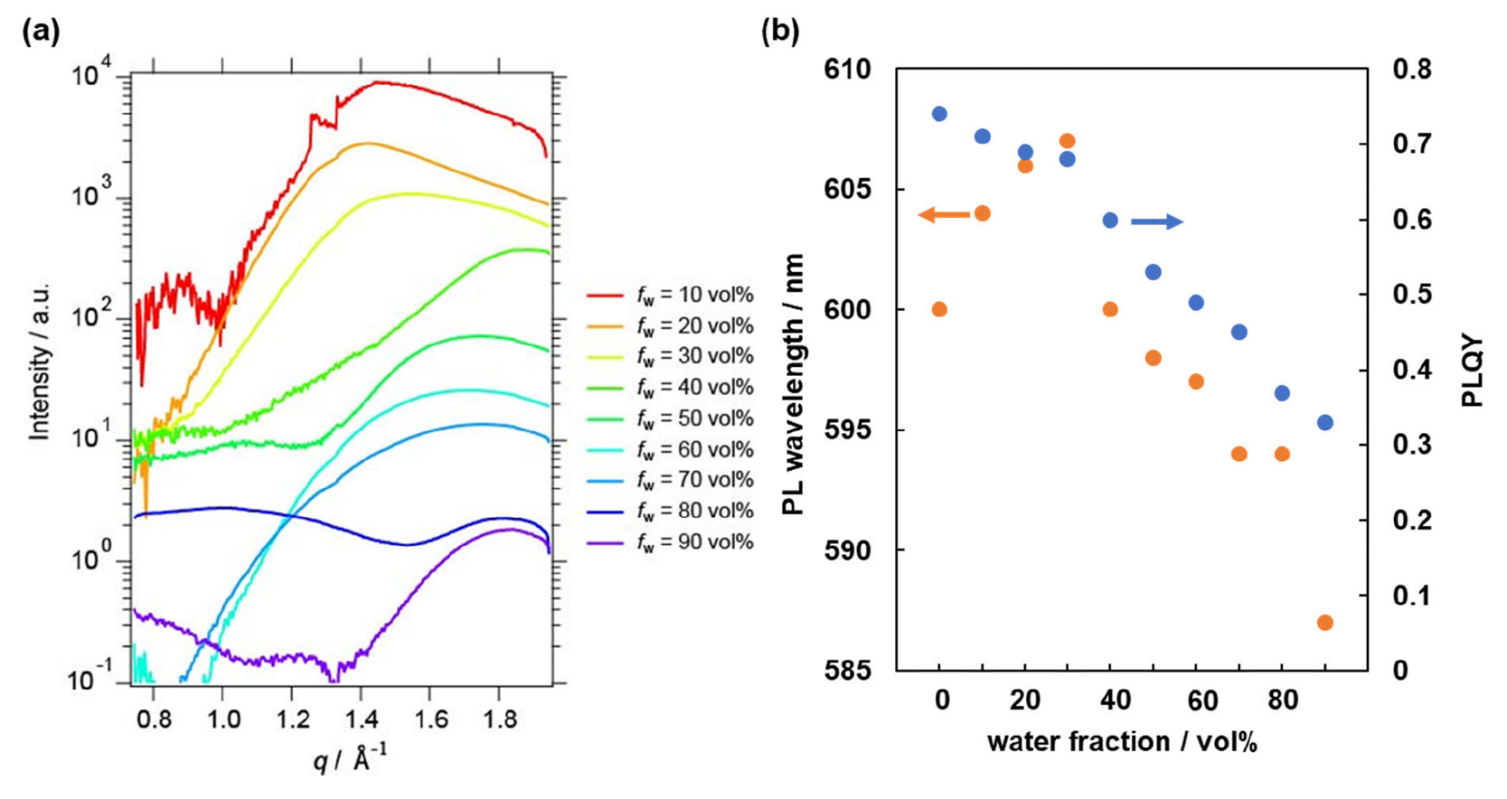
WAXS and PL measurements of Cz-COPV2-BTz-COPV2-Cz solutions in water–THF: (a) scattering profiles of solutes obtained by WAXS measurements at room temperature and (b) PL wavelength (orange) and PLQY (blue) as a function of the water content.

This aggregation behavior observed by WAXS measurements is consistent with that inferred from photoluminescence (PL) measurements. For a 1 μM solution of Cz-COPV2-BTz-COPV2-Cz, the PL maximum wavelength in THF appeared at 600 nm and gradually shifted bathochromically to 607 nm with increasing water fraction up to *f*_w_ = 30 vol % (orange dots in Figure 3b), indicating an increase in the polarity of the surrounding environment. At *f*_w_ = 40 vol %, the peak wavelength abruptly shifted back to 600 nm, and further increasing the water fraction at *f*_w_ = 90 vol % induced a hypsochromic shift of the peak to 587 nm. This shift suggests a decrease in the ambient polarity of a fluorophore, which can be attributed to the formation of a hydrophobic environment as a result of the aggregation of solute molecules.^37,39,40^ The significant decrease in the PLQY from 0.68 at *f*_w_ = 30 vol % to 0.60 at *f*_w_ = 40 vol % corroborates the occurrence of intermolecular interactions (blue dots in Figure 3b). The ACQ becomes more prevalent upon further increasing the water content, ultimately converging toward the PLQY value of the solid state (0.26).

To combine these measurement results and discuss the water fraction dependence of the aggregation behavior of the hydrophobic dye molecules, it should first be noted that the characteristics and observable range of each measurement (DLS, SANS, and WAXS) are different from each other; i.e., they afford complementary information. The SANS measurements afford information on the size of the dye and dye/THF aggregate structures in the range of 0.03 ≤ *q* ≤ 0.3 nm^–1^, corresponding to 21–209 nm in real space. The WAXS data afford information on the distance between the dye molecules in a spatial range from 0.3 to 1 nm. Considering these conditions and experimental data, we would like to propose the model outlined in Figure 4.

**Figure 4:**
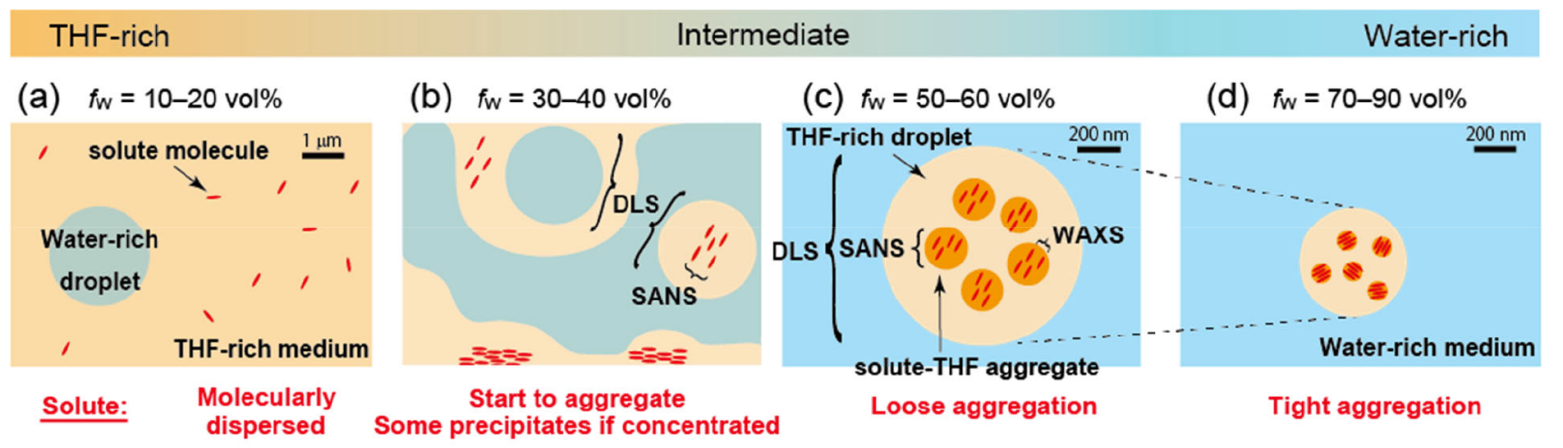
Schematic illustration of the solvent mixture ratio dependence of the aggregation of Cz-COPV2-BTz-COPV2-Cz in water–THF. The size of the solute molecules is drawn larger than it actually is.

(a) In the THF-rich region (*f*_w_ = 10–20 vol %), the solute molecules (Cz-COPV2-BTz-COPV2-Cz; Figure 4a) are dispersed individually. It seems feasible to assume that the objects observed by DLS are water-rich droplets.

(b) In the early intermediate region (*f*_w_ = 30–40 vol %; Figure 4b), the solubility of Cz-COPV2-BTz-COPV2-Cz decreases abruptly and precipitates form at a concentration of 10 μM. Dissolved Cz-COPV2-BTz-COPV2-Cz molecules are present as aggregates sized ∼100–120 nm, where dye molecules do not interact strongly with each other,^27^ as indicated by the absence of any PL quenching. Meanwhile, THF-rich regions were not formed according to the results of the SANS measurements. This primary aggregation behavior is caused by an increase of the aqueous environment that limits the space where hydrophobic solutes can exist within the mixed solvent system. At room temperature, a THF content of 10–40 mol % in water, which corresponds to a *f*_w_ of 25–65 vol %, is located close to the phase separation boundary in the phase diagram^17,21^ of the water–THF binary system, and there can be a composition fluctuation in the system that induces the non-solvency of the hydrophobic solute in the water–THF mixed solvent.^21^

(c) In the late intermediate region (*f*_w_ = 50–60 vol %), the miscibility of the hydrophobic solute in the water–THF mixed solvent increases again. The results of the DLS measurements suggest the presence of μm-scale objects, as estimated from the relaxation time, while the Guinier approximation of the SANS profiles suggests the presence of objects with a size of <100 nm. The scattering intensity distribution obtained from the DLS measurements is strongly affected by the presence of particles whose size is comparable to the wavelength of light. In the Guinier approximation, the radius of gyration is underestimated when the anisotropy of the particles is high. Nevertheless, the large discrepancy between the DLS and SANS results indicates the presence of a hierarchical structure. Supporting experiments afforded further insight into this issue: Filtration of the Cz-COPV2-BTz-COPV2-Cz solution in the water–THF mixed solvent (*f*_w_ = 50 vol %) using a 450 nm polytetrafluoroethylene (PTFE) filter afforded a colorless liquid that did not show any fluorescence, while fluorescent material remained on the filter (Figure S2 of the Supporting Information), suggesting that the fluorescent dye molecules are incorporated in aggregates whose size is >450 nm. On the basis of the measurements and the filtration experiment, we postulate the hierarchical structure shown in Figure 4c. Thus, the solute–THF aggregates with tens of nanometers in size (as evident by SANS) are present on the surface or inside larger objects of ∼1000 nm in size (as evident by DLS and microscopy). At this point, we assume that the larger objects are THF-rich droplets, which have previously been proposed for a similar hierarchical structure using another solute in water–THF in the low THF-loading regime.^22^ In the solute–THF aggregates, Cz-COPV2-BTz-COPV2-Cz molecules experience some but not severe intermolecular interactions (loose aggregation), which is consistent with the observed PLQY (>0.50) that is much higher than that of the solid state.

(d) A further increase of the water fraction decreases the sizes of the objects observed by DLS and SANS, and the intermolecular distance between molecules of Cz-COPV2-BTz-COPV2-Cz decreases, as suggested by the results of the WAXS measurements. This change can be interpreted in terms of a once more increasing miscibility between THF and water, which results in a gradual desolvation of THF from the solute–THF aggregate as the water fraction further increases, while the hydrophobic Cz-COPV2-BTz-COPV2-Cz molecules remain there. As a result, in the water-rich region (*f*_w_ = 70–90 vol %; Figure 4d), Cz-COPV2-BTz-COPV2-Cz molecules are forced to form tighter aggregates, wherein considerable intermolecular interactions between the fluorescent molecules occur, which results in significant fluorescence quenching.

In summary, we investigated the formation of aggregates composed of THF and Cz-COPV2-BTz-COPV2-Cz molecules as well as their size changes depending upon the water fraction. The role of amphiphilic THF as a medium and a promoter of hydrophobic Cz-COPV2-BTz-COPV2-Cz in the ternary system was elucidated on the basis of the physicochemical data. The obtained results in their entirety suggest that solute aggregates start to form at *f*_w_ ≈ 30 vol % and their size depends upon the water fraction. Moreover, the relationship between the aggregate size and the luminescence properties was examined. However, it should also be noted here that the simultaneous formation of other structures, such as nanobubbles and THF hydrates, cannot be ruled out in this stage. Nevertheless, the present model can be expected to be helpful in controlling the aggregate size and density toward achieving desired properties in a broad range of applications, such as the precise control of the dispersion of emissive dye in a matrix for light-emitting devices and the control of carrier size and drug molecule dispersion in drug delivery systems.

## Methods

*General Procedures*. The fluorescent material Cz-COPV2-BTz-COPV2-Cz was prepared according to literature procedures.^37^ Perylene and tetraphenylethene were purchased from Fujifilm-Wako Pure Chemical Co. 1,1,2,3,4,5-Hexaphenylsilole and [[(3,5-dimethyl-1*H*-pyrrol-2-yl)(3,5-dimethyl-2*H*-pyrrol-2-ylidene)methyl]methane](difluoroborane) were purchased from Sigma-Aldrich and Tokyo Chemical Industry Co., respectively. These commercially available fluorescent materials were used without purification. Mill-Q water was used for all measurements. The following solvents were purchased commercially and used without purification: THF (spectroscopic grade, without a stabilizer, Fujifilm-Wako Pure Chemical Co.), THF-*d*_8_ (>99.5% D, Merck), and D_2_O (99.9% D, Sigma-Aldrich). For the measurements, the solute was dissolved in THF, before water was added in one shot to make a solution of the desired ratio. The concentration of the solution was approximately 1–10 μM.

*Instruments*. Fluorescence spectra were recorded on a JASCO FP-6500 spectrophotometer. PLQY values were measured on a Hamamatsu Photonics C9920-02 absolute PL quantum yield measurement system, and absolute quantum yields were determined using a calibrated integrating sphere system. DLS profiles were measured on an Otsuka Electronics ELSZ-2000ZS particle size analyzer. Fluorescence microscopy images were recorded on a Keyence BZ-9000 fluorescence microscope with the exposure of 0.25 s.

*SANS Measurements*. SANS measurements were carried out using a SANS-J diffractometer at the Japan Research Reactor-3 (JRR-3) of the Japan Atomic Energy Agency (JAEA) in Tokai, Japan.^41^ The incident neutron beam was monochromated using a velocity selector with a wavelength (λ) of 0.65 nm and a wavelength distribution (Δλ/λ) of 0.15. The incident beam size was defined using a 20 × 20 mm^2^ aperture 10 m upstream of the samples and a 15 mm diameter aperture at the sample position. Two two-dimensional (2D) position-sensitive detectors were used to detect scattered neutrons from the sample. The larger detector was 650 × 680 mm (length × width) and was composed of 95 ^3^He tube detectors (diameter of 8 mm), and the smaller detector was 650 × 385 mm (length × width) and composed of 48 ^3^He tube detectors (diameter of 8 mm). Two sample–detector distances (*L*) of the larger detector were set as 4 and 10 m along the beam path of the incident neutrons, while that of the smaller detector was set in a high-angle region to the beam path of the incident neutrons with *L* = 0.96 m. These instrumental configurations enabled us to cover a *q* region of 0.04 < *q* < 6 nm^–1^. The 2D scattering data recorded on these two detectors were corrected by counting efficiency before azimuthal averaging was applied to obtain the scattering intensity distribution as a function of *q*. Instrumental background and air scattering were subtracted as well as the corresponding empty cell scattering for each scattering curve. Absolute intensity units (cm^–1^) were obtained using an Al plate as a standard reference. The corrected scattering intensity distribution was designated as *I*(*q*).

*WAXS Measurements*. WAXS measurements were carried out at BL10C of the Photon Factory at the High Energy Accelerator Research Organization (KEK), Japan, using a PILATUS3 200K detector (Dectris, Ltd.), an X-ray wavelength of 1.5 Å, and a sample–detector length of 0.26 m. Measurements were performed at room temperature (24 ± 1 °C). The length and tilt angle of the detectors were precisely calibrated using the data of a standard sample (silver behenate). After calibration, circular averages for the obtained 2D images were recorded to generate one-dimensional diffraction profiles. To elucidate the aggregate structure of Cz-COPV2-BTz-COPV2-Cz in THF–water, WAXS measurements were performed for the ternary system and the THF–water binary system. The results of the binary mixture were subtracted from those of the ternary mixture to obtain the scattering profile of Cz-COPV2-BTz-COPV2-Cz.

## Supporting Information Available

DLS charts and pictures of the filtration experiment (PDF)
