# Supplementary material for: Water Fraction Dependence of the Aggregation Behavior of Hydrophobic Fluorescent Solutes in Water–Tetrahydrofuran
Source: J Phys Chem Lett. 2023 Dec 7;14(49):11235–41. doi: 10.1021/acs.jpclett.3c02882 (PMC10726388; doi:10.1021/acs.jpclett.3c02882)
Supplement: Supplementary file 1 — jz3c02882_si_001.pdf [file jz3c02882_si_001.pdf]

**Supporting Information**

# Water-fraction dependence of the aggregation behavior of hydrophobic fluorescent solutes in water–tetrahydrofuran

Hayato Tsuji,[1*] Masaki Nakahata,[2] Mafumi Hishida,[3] Hideki Seto,[4] Ryuhei Motokawa,[5] Takeru Inoue,[1] and Yasunobu Egawa[1]

[1] Department of Chemistry, Faculty of Science, Kanagawa University, 3-27-1 Rokkaku-bashi, Kanagawa-ku, Yokohama 221-8686, Japan

[2] Department of Macromolecular Science, Graduate School of Science, Osaka University, 1-1 Machikaneyama-cho, Toyonaka, Osaka 560-0043, Japan

[3] Department of Chemistry, Faculty of Science, Tokyo University of Science, 1-3 Kagurazaka, Shinjuku, Tokyo 162–8601, Japan

[4] Institute of Materials Structure Science, High Energy Accelerator Research Organization, Tokai, Ibaraki 319-1106, Japan

[5] Materials Sciences Research Center, Japan Atomic Energy Agency, Tokai, Ibaraki 319-1195, Japan

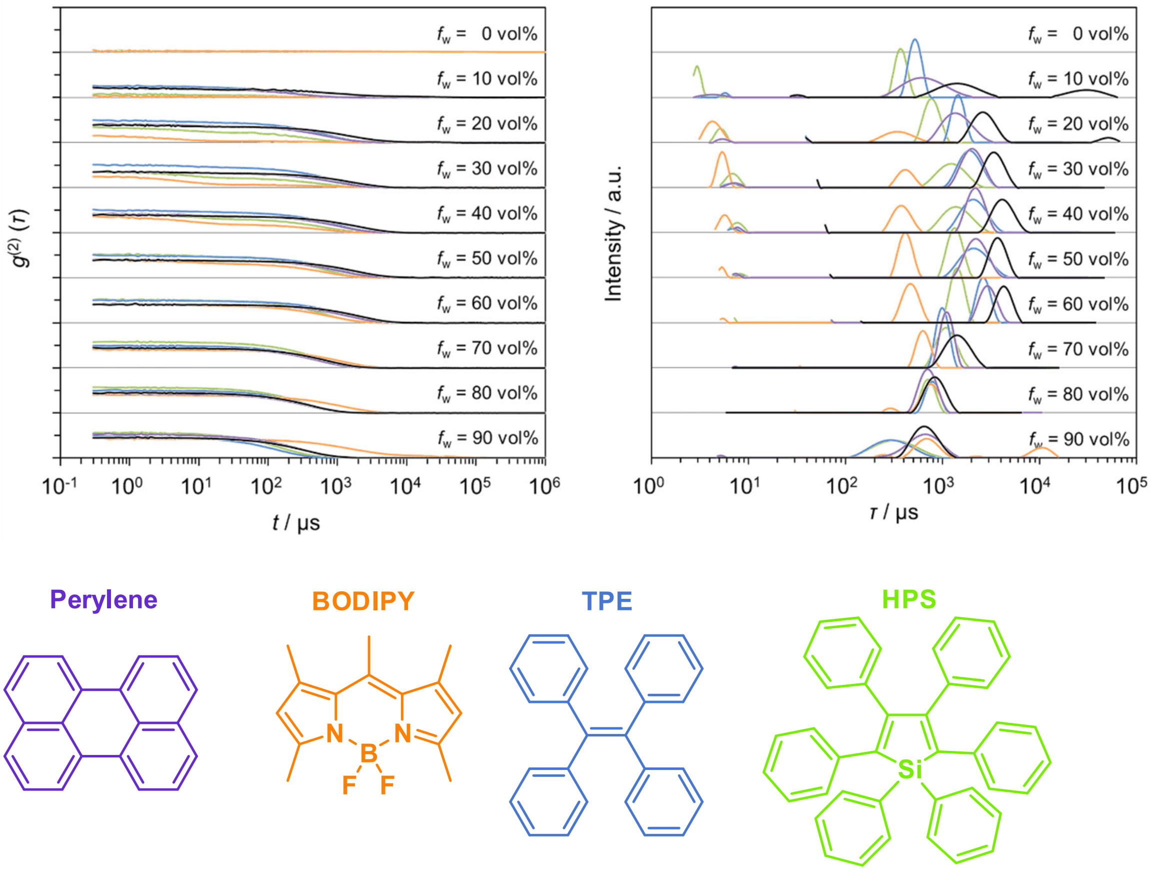


**Figure S1** Water fraction dependence of the DLS profile of different fluorescent solutes. Perylene, BODIPY, TPE, and HPS, and solvent only.

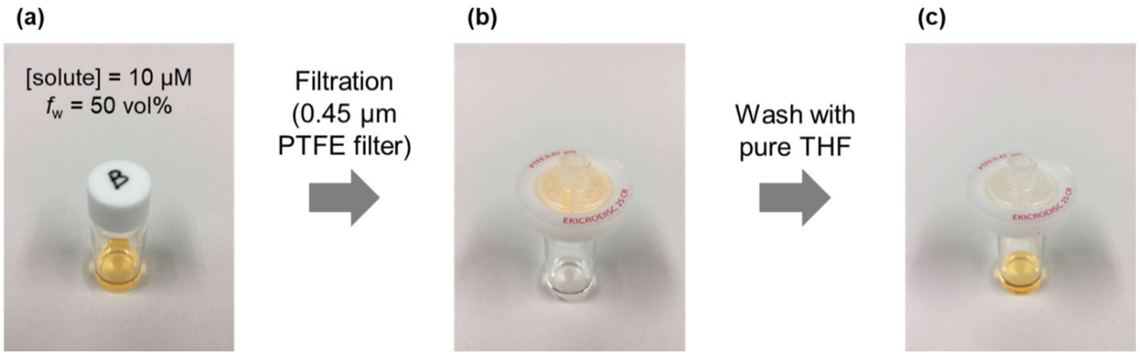


**Figure S2** Filtration experiment of Cz-COPV2-BTz-COPV2-Cz in water/THF = 1/1 mixed solvents. (a) Before filtration, (b) after filtration through a 0.45-μm PTFE filter, and (c) after washing the filter with pure THF.
